# Tritosomes-Digestion for LC-MS Conjugated Payloads Quantitation: A Universal Approach for Dual-Payloads ADCs

**DOI:** 10.3390/ijms27135874

**Published:** 2026-06-29

**Authors:** Francesco Molinaro, Gabriele Sergio Colangelo, Patrizia Cocco, Andrea Di Ianni, Diana Knapp-Buehle, Andrea Paoletti, Elisa Bertotti, Kyra Cowan, Federico Riccardi Sirtori, Luca Barbero

**Affiliations:** 1 Innovative Bioanalytics, New Biological Entities, Drug Metabolism and Pharmacokinetics (NBE-DMPK), Research and Development, Merck Healthcare KGaA, RBM S.p.A.—Istituto di Ricerche Biomediche “A. Marxer”, an Affiliate of Merck KGaA, Via Ribes 1, 10010 Colleretto Giacosa, TO, Italy; gabriele-sergio.colangelo@merckgroup.com (G.S.C.); patrizia.cocco@merckgroup.com (P.C.); andrea.di-ianni@merckgroup.com (A.D.I.); andrea.paoletti@merckgroup.com (A.P.); elisa.bertotti@merckgroup.com (E.B.); federico.riccardi-sirtori@merckgroup.com (F.R.S.); 2 New Biological Entities, Drug Metabolism and Pharmacokinetics (NBE-DMPK), Research and Development, Merck Healthcare KGaA, Frankfurter Strasse 250, 64293 Darmstadt, Germany; diana.knapp-buehle@merckgroup.com (D.K.-B.); kyra.cowan@merckgroup.com (K.C.)

**Keywords:** antibody-drug conjugates, dual-payload, tritosome, bioanalysis, conjugated payload

## Abstract

Bioanalytical methods to quantitate conjugated payloads are essential for assessing antibody-drug conjugate (ADC) stability and pharmacokinetics (PK). Dual-payload ADCs present analytical challenges; different linker chemistries can require complex digestion conditions to perform the cleavage. Developing separate methods for each linker combination can be time and resource demanding. Rat tritosomes—purified lysosomal fractions from Triton-treated rat liver—provide a comprehensive enzymatic mixture that mimics the lysosomal environment. The presented bioanalytical method combines immunoaffinity purification with tritosome-mediated digestion for simultaneous quantitation of dual-conjugated payloads. The method was applied to a model dual-payload ADC containing two different cytotoxic payloads, conjugated using different enzymatically cleavable linkers, with an unrelated DAR (drug-to-antibody ratio). Method validation in mouse plasma demonstrated excellent accuracy (bias ± 20%, LLOQ and ULOQ ± 25%) and precision (coefficient of variation CV% ≤ 20%, LLOQ and ULOQ ± 25%) across all concentration levels (lower to upper limit of quantitation, LLOQ to ULOQ) for both payloads, with 100% of quality control samples (QCs) meeting acceptance criteria for hybrid LC-MS/MS quantitation methods. This tritosome-based approach provides a unified, efficient platform for multi-payload ADC bioanalysis, eliminates linker-specific method optimization, and enables robust support for preclinical studies. The method has been tested for accuracy and precision on 4 different model ADCs and employed to quantify the conjugated payloads in in vivo samples from a homozygous hFcRn transgenic mouse model (Tg32) PK study, resulting in reliable data in accordance with total antibody measurements.

## 1. Introduction

Antibody–drug conjugates (ADCs) couple the specificity of monoclonal antibodies with potent small-molecule payloads via engineered linkers. They enable targeted tumor cell killing while aiming to limit systemic exposure compared to conventional chemotherapy. This results in an increased therapeutic window and improved patient compliance [1,2]. Rapid advances in payloads, linkers, and conjugation chemistries have expanded the ADC design space and motivated strategies to overcome resistance and tumor heterogeneity [3].

Dual-payload ADCs represent the next step. They co-deliver two distinct drugs to the same target cell to achieve complementary or synergistic mechanisms, mitigate cross-resistance, and potentially widen the therapeutic index. In these constructs, site-specific, homogeneous conjugation and careful selection of payload pairs and ratios are required to balance efficacy and toxicity. While multiple dual-payload designs show enhanced activity in preclinical models, clinical validation is still pending, underscoring the need for robust translational frameworks to guide development [4,5].

Linker architecture and controlled intracellular cleavage are central to ADC performance. Enzyme-sensitive peptide linkers (e.g., cathepsin B–labile) are designed to remain stable in circulation and cleave post-internalization within lysosomes to trigger payload release; this paradigm is widely implemented across approved and clinical-stage ADCs [2]. Beyond chemistry, linker length and the conjugation microenvironment can modulate stability and release kinetics. Shorter linkers may be sterically shielded by the antibody, thereby improving in-circulation stability, and—given identical linkers—the conjugation site can alter release kinetics detectable in assays of antibody-conjugated payload [6]. Notably, for some constructs (e.g., vcMMAE-based ADCs), cathepsin B cleavage itself may be relatively insensitive to drug location or antibody carrier, highlighting context-dependent effects of conjugation position [1].

Sensitive bioanalytical methods are crucial for quantifying conjugated payloads, characterizing linker stability and cleavage, and defining pharmacokinetics across the discovery, preclinical, and clinical stages. For cleavable linkers, targeted liquid chromatography coupled to mass spectrometry (LC–MS/MS) assays that enzymatically release the payload ex vivo (e.g., using purified cathepsin B for Val-Cit linkers) enable the selective measurement of antibody-conjugated payload and complement immunoassays for total antibody and intact ADC, as well as assays for free drug and metabolites [7,8,9,10,11,12].

In dual payload ADCs, bioanalytical characterization must further resolve each payload’s conjugated and unconjugated forms, potential inter-payload interactions, and drug-to-antibody ratio (DAR) distributions across conjugation sites, all while maintaining assay selectivity and avoiding cross-interference. These requirements place a premium on orthogonal methods—combining targeted LC–MS/MS (with enzymatic or chemical release), immunocapture workflows, intact and subunit mass spectrometry, and cell-based functional readouts—to ensure accurate quantitation and mechanism-aware interpretation across discovery, preclinical development, and clinical translation [6,7,8,11].

This work focuses on dual-payload ADCs and presents strategies for setting up and validating a bioanalytical method to quantify dual-conjugated payloads in preclinical matrices. The method defines the analytes and species to be measured, implements enrichment and controlled release (followed by LC–MS to distinguish each payload and its metabolites), and performs validation tests to assess selectivity, sensitivity, precision, matrix effects, and dilution linearity. The present work introduces, for the first time, lysosomal enzymatic cleavage of linkers applied to in vivo samples for quantitation purposes. Existing quantitation methods dedicated to the conjugated payload focused on optimizing linker molecular specificities, like valine-citrulline linkers cleaved by cathepsin B or papain [13,14] and ß-glucuronide linkers cleaved by ß-glucuronidase enzyme [15]. Choosing the appropriate enzyme for linker payload hydrolysis is essential, as it directly influences the optimization of digestion parameters necessary for efficient payload release. Considering a multiple payload release reaction from a single ADC, the number of variables to consider can complicate assay development. The use of lysosome extracts as an in vitro enzymatic machinery for linker-payload cleavage may help target various linker-payload constructions with different chemistries, as well as antibody fragmentation to enhance payload release [16]. The proteolytic machinery in lysosomal extracts does not require the fine-tuning of enzymatic concentrations, as protein digestion occurs in high-quality and ready-to-use organellar microenvironments that mimic in vivo protein degradation. Lysosome extracts can be contaminated by other organelles, which may affect the proteolytic machinery and the digestion yield of therapeutic proteins [17]. Strategic interventions on lysosomal bilayer composition after the administration of less dense polymers (such as Triton WR1339 or Tyloxapol) in living rats have generated specific lysosomes, named tritosomes, characterized by a distinct lipid profile and lower density. This feature facilitates the easier separation of lysosomal vesicles from other isopycnic organelles (such as mitochondria and peroxisomes) in discontinuous density gradient solutions, which provides a less contaminated final product for metabolic stability and pharmacokinetic/pharmacodynamic (PK/PD) analysis [18]. To initiate method set up, the choice between human lysosome extracts and rat tritosomes as lysosomal machinery was guided by different factors. As a first main consideration, the standardization of tritosomes using Sprague-Dawley rats was preferred over human lysosome extracts due to the need for human donors and reduced pool availability. Tritosomes afford a more consistent extract composition, making them suitable to satisfy critical reagent requirements in late stages of biological methods and avoiding batch-to-batch variability. Cost considerations gave further reasons for the selection of tritosomes over human-derived lysosomes as a balanced choice from a project budget management perspective. Human lysosome extracts have the advantages of presenting human-specific enzymatic isoforms, maintaining the physiological lipidome, and mimicking clinical behavior. However, these advantages are offset by lower purity regarding cell contaminants such as mitochondria or peroxisomes, which can cause interference with payloads. On the contrary, the presence of Tyloxapol in rat liver tritosomes is not physiological, but allows for obtaining an increased purity of the lysosomal extract, together with an enzymatically enriched environment and a higher level of standardization. The use of Tyloxapol for the separation minimizes interference from cellular contaminants, while the enriched enzymatic environment provides high concentrations of cathepsins and nucleases, ideal for high-yield digestion. Taking this into consideration, the independence from human sources allows for more easily obtaining lysosomal fractions from a larger pool of animals instead of a reduced number of human donors, providing a much better purified matrix combined with reduced risk of cellular contaminants that could provide unexpected interferences or interactions with the liberated payloads.

Integrating the lysosomal step into dual-payload ADCs degradation can ensure that payload quantitation reflects intracellular mechanisms through biologically relevant processing and time-resolved kinetic profiling rather than relying on single enzyme cleavages. Together, these features demonstrate that lysosomal enzymatic release can broaden applicability. This enables quantitative analysis of dual-payload ADCs bearing diverse cleavable linkers and supports more mechanistically faithful PK/PD interpretation and design optimization [4]. This method was validated for linearity, accuracy, precision, sensitivity, selectivity, matrix effect, carryover, recovery, and sample dilution on a tool ADC (ADC A) bearing enzymatically linked the payload A (DAR X) and a different enzymatically linker attached to payload B (DAR Y) to explore robustness and reliability. Tests related to the single payloads, as were already explored in-house in previous studies about the specific small molecules, were not repeated as they were out of the scope of this work. Then it has been verified for accuracy and precision to a total of 4 tool ADCs with the same linker payloads and DARs on different conjugation sites.

## 2. Results

**Figure 1:**
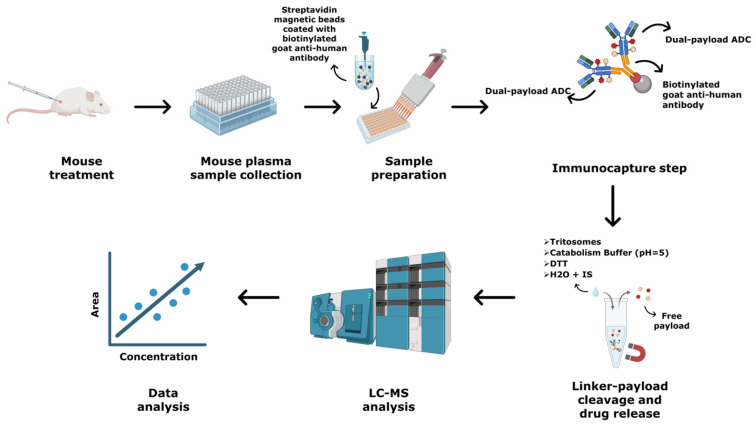
Schematic representation of conjugated-payload quantitation workflow from dual-payload ADC tools in mouse plasma samples. Samples from relevant PK species (Tg32 mouse models) were collected in storage tubes. Streptavidin magnetic beads were functionalized with an appropriate capture reagent (e.g., biotinylated goat anti-human IgG Fcγ fragment specific) and mixed with aliquots of the samples. After the immunocapture step, a tritosome preparation was applied directly to the magnetic bead suspension and incubated overnight at 37 °C. The reaction mixture underwent protein precipitation in an organic solvent, discarding a protein pellet enriched with lysosomal enzymes. The supernatant was evaporated, and the payloads were resuspended under acidic conditions before LC-MS/MS quantitation. Finally, data from LC-MS analysis were analyzed using dedicated statistical software. The figure was generated using BioRender.com, Colangelo, G. S. (2026) https://BioRender.com/8r06a85, accessed on 27 June 2026.

A multi-analyte method has been developed, resulting in reproducible and reliable quantitation for both payloads. The method uses a unique digestion for both the linkers, employing rat liver tritosomes. The method was solid and applicable to a high-throughput parallel procedure using the 96-well format, satisfying acceptance criteria in the validation tests and being applied to in vivo samples derived from a PK study. The final protocol employed 3 µL of mouse plasma sample to perform immune purification using a generic anti-human capture, followed by washing steps to isolate the circulating human-antibody-based ADC from plasma contaminants and free payload. Tritosome-based digestion was then performed on the purified ADC to let the enzymes cleave the different linkers. After overnight digestion incubation, protein precipitation by organic solvent, evaporation, and resuspension were applied to submit the samples to LC-MS detection. The complete protocol is described in the materials and methods and depicted in Figure 1.

### 2.1. Method Validation

#### 2.1.1. Method Validation Results

Method validation results met the acceptance criteria internally applied for hybrid bioanalytical methods to quantitate each conjugated payload in a range of ADC concentrations from 5.0 to 1000 nM.

Main parameter results for each of the payloads are reported in Table 1.

#### 2.1.2. Recovery Result Evaluation

**Figure 2:**
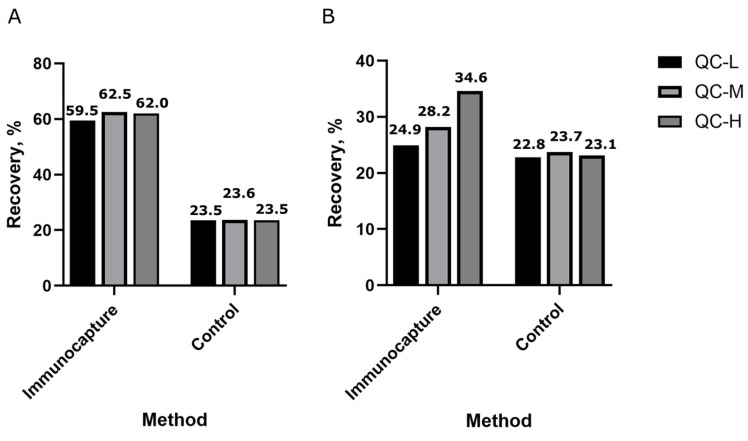
Recovery % of Payload A (**A**) and Payload B (**B**) analytes in immunocapture and quality control samples at each tested concentration level. Data represent *n* = 3 independent replicates.

**Figure 3:**
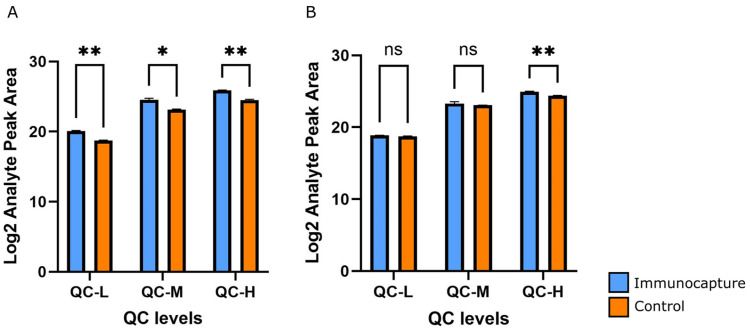
Analysis of the effect of the immunocapture step on analyte signals across QC samples. Multiple unpaired *t*-test analysis per QC level was performed comparing analyte signals between immunocapture and control samples. (**A**): *t*-test analysis on payload A peak area showed significant differences across all QC-levels, proving the effect of immunocapture on increasing analyte signal compared to control. (**B**): *t*-test analysis on payload B peak area across all QC samples only revealed significant differences at high concentrations (QC-H), whereas no significant variation was highlighted in low and medium concentrations (QC-L and QC-M). The results are from *n* = 3 independent measurements. According to *p*-values, the level of significance (*p*-value) is as reported: ns > 0.05; * <0.05; ** <0.01.

Recovery was further explored to understand the impact of digestion and immunocapture for each analyte. Overall method recovery was reproducible, with CV% below the 20% acceptance criteria (Table 2). The obtained areas were reproducible across validation runs and tests, providing constant values of area ratio with the internal standard. Additional investigation into analyte recovery was conducted across different concentration levels to determine whether immunocapture or digestion effects may cause discrepancies in analyte signals. Recovery % values were lower without the immunocapture step, with a marked effect observed for the conjugated payload A analyte (Figure 2A). Comparative analysis across quality control levels showed a significant difference (*p*-value < 0.05) between immunocaptured samples and their relative counterparts at each level. A multiple unpaired *t*-test analysis per QC level confirmed that the immunocapture step enhanced peak areas, regardless of analyte concentration, compared with control sample areas (where no immunoaffinity was performed). These results can be related to the excess of protein background in the control samples, which is likely to interfere with the activity of lysosomal hydrolytic enzymes on the targeted drug. Therefore, the broader activity of specific classes of enzymes on matrix substrates can significantly reduce the release of the payload linked via enzymatically cleavable linkers, depending on the enzyme class. In addition, the lower signals from payload A across QC levels in control samples without immunocapture might be due to matrix interference in the electrospray ionization (ESI) source, leading to a drastic drop in analyte signal during the detection phase. On the other hand, the results related to payload B showed a less marked difference in the condition with and without immunocapture (Figure 2B). This was likely due to different abundance and turnover of enzymes in the presence of the matrix-interfering substrates, resulting in outcomes closer to those of the immunopurified samples. Matrix-interference effects did not effectively impact payload B signals, given that the %yield of payload B in control samples was in line with %recovery in immunocapture samples. Payload B also displayed fluctuations in the recovery score across QC levels in the immunocapture samples, with increasing values in line with concentration. However, the coefficient of variation (CV%) of the recovery score was below 20% and within the acceptance criteria. Conversely, the same trend is not observed in the control samples without immunocapture, where payload B recovery remains constant at 23%, and no concentration-dependent effects were observed. As a result, the consistency of the recovery values across QC levels demonstrated that the method remains fit-for-purpose for PK applications. Sample drug isolation via immunocapture also showed a significant increase in payload B signal in high concentration (H) QC levels. In contrast, no significant signal enhancement (*p*-value > 0.05) was observed in low and medium (L and M) QC samples (Figure 3).

### 2.2. Method Application to In Vivo PK Samples

The method was tested for accuracy and precision and applied to in vivo PK samples on 4 different test ADCs, here named from letter A to D, with different conjugation positions but constant nominal DAR (payload A = X, payload B = Y). X and Y values refer to DAR values of payload A and B attached to a single antibody structure, respectively. All dual-payload ADCs under evaluation shared the same DAR value linker payloads but were realized using different conjugation strategies. Applicability for each tested ADC was verified on reproducibility and linearity, evaluating a standard calibration curve (range 5–1000 nM in terms of ADC) and 5 replicates on 5 concentration levels. In terms of sensitivity, this meant an LLOQ sensitivity of the method below 10 ng/mL in terms of conjugated payload for both payloads. While for ADC A an extended validation was performed, covering multiple tests, condensed results for ADCs from B to D are shown in Table 3. In vivo PK samples deriving from a hFcRn Tg32 mouse study were previously analyzed by ligand binding assay for total antibody quantitation.

**Figure 4:**
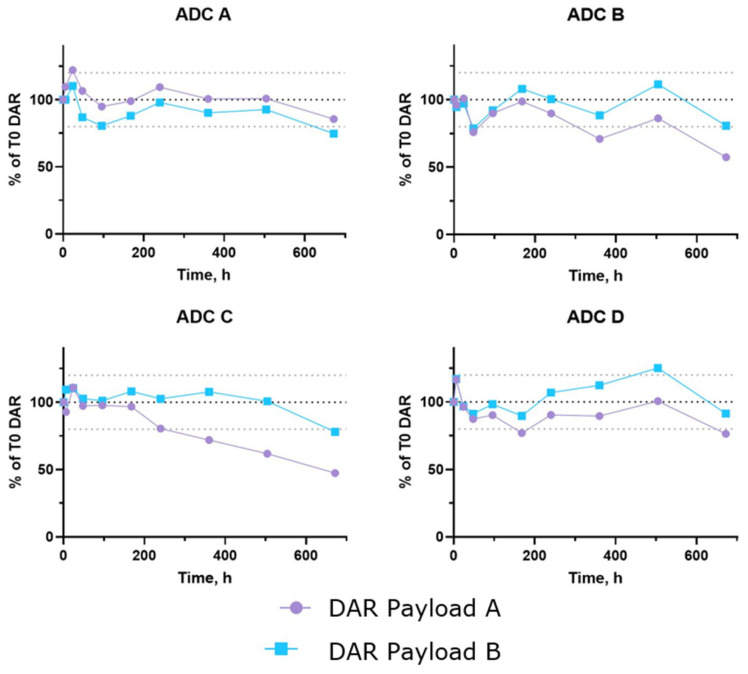
Normalized DAR as % of first timepoint results for tested ADCs (A, B, C, D) in PK study in hFcRn Tg32 mice, dotted lines mark the +/−20% variability from the t0 value, considered as 100%.

Pharmacokinetic profiles obtained from the conjugated payload quantitation from the 4 different ADCs were consistent with the total antibody concentrations obtained by the ligand binding assay. Analytical method sensitivity allowed for following the PK profile up to the last sampling time (672 h). The quantitative analysis allowed drug-to-antibody ratio monitoring (DAR) during the entire PK experiment and the assessment of the stability of the linker payload for the entire study. The ratio between conjugated payload and total antibody calculated at 0.167 h was considered as the initial DAR value, then the in vivo DAR vs. time variation was monitored, providing precious information on ADC in vivo stability, which will be used to rank the candidates and the conjugation strategies. Normalization reduced potential BIAS effects between orthogonal methods and allowed us to explore how the DAR evolved over time (Figure 4).

## 3. Discussion

An innovative methodology has been described, which allows a universal approach to cleavable linker ADCs for LC-MS-conjugated payload quantitation. The intrinsic nature of linker payloads developed for ADCs requires that the mechanism for payload release in lysosomes occur naturally to exert the warhead effect in cells. The present protocol simplifies sample preparation and mimics the lysosomal environment and reactions, releasing the payload for quantification. The method demonstrated robustness and reliability in quantifying two different payloads conjugated with different linkers simultaneously. In this context, lysosome extracts provide the environment needed for the cleavage of multiple enzyme-targeted linkers. Moreover, the immunocapture step increased the yield of conjugated payload, reducing the complexity of the substrate for digestion and eliminating the need to quantify free payload. In the absence of immunocapture, abundant proteins such as albumin and endogenous immunoglobulins may saturate the activity of lysosomal hydrolases. This interference can ultimately reduce cleavage efficiency and payload release, leading to abrupt scaling down of the payload signals. Moreover, higher matrix load could increase LC–MS/MS ion suppression, which can be reduced using the immunocapture step. All validation parameters were satisfied, thereby presenting this solution as a potential universal sample preparation protocol for quantitation of conjugated payloads or multiple conjugated payloads, reducing the number of experiments and the time required for single or combined digestions.

## 4. Materials and Methods

### 4.1. Animals

The huFcRn Tg32 homozygous mice (Strain: B6.Cg-Fcgrttm1Dcr Tg(FCGRT)32Dcr/DcrJ) were purchased from Charles River Laboratories Italia S.r.l. (Calco, Italy). The study was carried out at RBM spa in accordance with Italian law No. 26 of 4 March 2014 (implementation of Directive 2010/63/EU) and Merck KGaA animal welfare policy. RBM spa is fully authorized by the Italian Ministry of Health to run in vivo studies (protocol No. 18ECB.31). Mice were housed in a 12:12 h light–dark cycle with unrestricted access to food and water, 20–22 °C, 45–65% humidity conditions. The cages were appropriately enriched and in a static condition.

### 4.2. In Vivo Tg32 PK Study

Mouse plasma samples were obtained from an in vivo pharmacokinetic (PK) study designed to evaluate the PK profile of four antibody-drug conjugates (ADCs; A, B, C, and D). The study consisted of four experimental groups (one per ADC); no vehicle control group was needed since the goal of the study was to compare the PK properties of the different ADCs. The experimental unit was the individual animal. Each group comprised three animals (12 animals in total, 4 groups, *n* = 3 per group). The size of the experimental groups was determined to have 3 different PK profiles for each ADC to generate a robust dataset, also in case of two conflicting measurements. Treatment-naïve male and female huFcRn Tg32 mice, aged 5–7 weeks at the time of dosing and weighing 18–35 g, were used. Animals were allocated to treatment groups using a randomization method based on the bodyweight of the subjects. No specific strategy to further minimize the confounders was applied due to the limited size of the study. No animals were excluded from the study after a 4-day acclimatization period in which the health status of the subjects was checked at least 2 times per day. Blinding was not applied due to the nature of the study. ADCs were formulated in a buffer containing 10 mM histidine, 40 mM NaCl, 6% trehalose, and 0.05% Tween-20 (pH 5.5) and administered as a single intravenous injection via the tail vein at a dose of 3 mg/kg and a dose volume of 5 mL/kg. Injections were performed in awake animals restrained using a specific restraining device, with no anesthesia. Serial blood sampling was performed using a microsampling technique. Approximately 20 µL of blood was collected at the following points: 0.167, 6, 24, 48, 96, 168, 240, 360, 504, and 672 h post-dose. The total blood volume collected per animal was 200 µL (for the entire study) and was within recommended limits. Blood samples were centrifuged at 4 °C for 10 min at 2500× *g* to obtain plasma, which was stored at −80 °C until analysis. Total antibody concentrations in plasma were quantified using a ligand-binding assay. Plasma samples were subsequently pooled and analyzed for conjugated payload quantification. The concentration vs. time profiles were analyzed by Phoenix WinNonLin 6.3 to generate pharmacokinetic parameters.

### 4.3. ADC Molecule, Reagents, and Reference Animal Plasma Matrix

The internal “tools” ADC molecules (A, B, C, and D) were provided by the internal Merck KGaA conjugation laboratory. The four ADCs were all based on a human antibody conjugated with the same payloads A and B via, respectively, two distinct enzymatically cleavable linkers, requiring two different typologies of enzymes for their cleavage. The ADC A was used as a test molecule for method development and validation. Drug-to-antibody ratios of all the ADCs were determined by Reverse Phase (RP) LC-MS to verify the conjugation rate for both payloads.

A biotinylated immunocapture reagent employed in a magnetic bead-based purification step was Biotin-SP conjugated AffiniPure Goat Anti-Human IgG, Fcγ fragment specific (cat. 109-065-098, Jackson Immuno Research, West Grove, PA, USA). Streptavidin Mag Sepharose beads (GE28-9857-99, Cytiva, Marlborough, MA, USA) were coated with the biotinylated immunocapture reagent. Phosphate-buffered saline (PBS) for coating and immunocapture steps was acquired from Merck KgaA (cat. 18912-014, Merck KgaA, Darmstadt, Germany).

Sprague Dawley (SD) Rat Liver Tritosomes were used for the enzymatic digestion of ADCs and their relative linkers; Tyloxapol-treated, Mixed Gender, Pool of 160, 0.25 mL at 2.5 mg of protein per mL in a suspension medium of 250 nM Sucrose with 20 mM HEPES, pH 7.4 (cat. R0610.LT) were obtained by XenoTech, BIOIVT, and used together with associated 10× catabolism buffer (cat. K5200, XenoTech, BIOIVT, Kansas City, KS, USA). DL-Dithiothreitol (DTT) solution 1 M was purchased from Sigma Aldrich, now part of MilliporeSigma (St. Louis, MO, USA, cat. 646563).

C57 Mouse gender pooled plasma with K2EDTA as anticoagulant was obtained from BIOIVT and used for target molecule dilutions, calibration curve preparation, spiked samples, and as a reference matrix for control blanks.

LC-MS dedicated solvents, LC-MS grade Water, LC-MS grade Acetonitrile, Analytical grade Methanol, and analytical grade 2-Propanol (Sigma-Aldrich, Merck KGaA) were used for liquid-phase preparations, buffers, and solutions. Formic Acid 99% LC-MS grade (cat. 85048.051, VWR) was used as an acidic modifier for chromatography phases and resuspension solution. To normalize the payload steps and detection, deuterium-labelled versions of payload A and B were employed as internal standards (IS), provided by external suppliers. An aqueous internal standard solution with a final concentration of 50 ng/mL for both the IS molecules was used and applied in the digestion step.

### 4.4. Calibration Curves and Spiked Sample Preparation

Target molecules were diluted in mouse plasma matrix to a concentration of 1530 µg/mL ADC. The calibration curve was prepared starting from the 10 µM ADC solution in a range from 5 to 1000 nM concentration in terms of ADC, with 9 calibration standard points: 5, 10, 50, 100, 250, 500, 750, 900, and 1000 nM. Three additional concentration levels were chosen and obtained by independent dilutions in the mouse plasma matrix as QC checks with levels L (15 nM ADC), M (300 nM ADC), and H (800 nM ADC).

### 4.5. Mag Sepharose Beads Coating for Immunocapture

To prepare the immunocapture support and remove storage buffer, 15 µL of streptavidin magnetic bead slurry per sample was added to 35 µL of PBS buffer per number of samples (ex., per 10 samples: 150 µL of bead slurry added to 350 µL of PBS) and washed for 1 min on an inverter at room temperature. After mixing on an inverter, the beads were immobilized using a magnetic support, and the liquid phase was discarded and replaced with 50 µL per number of samples of PBS (ex., per 10 samples: 500 µL of PBS). After being removed from the magnetic rack and resuspending the beads, tubes were mixed on the inverter for 1 min at room temperature. This washing step was repeated to reach a total of 3 PBS washing steps. After the last washing step, beads were resuspended in 38 µL per number of samples of PBS (ex., per 10 samples: 380 µL of PBS) and 12 µL per number of samples of biotinylated immunocapture reagent (ex., per 10 samples: 120 µL of biotinylated immunocapture reagent). Tubes were mixed on the inverter at room temperature for two hours to allow immunocapture coating on the streptavidin magnetic beads. At the end of the 2-h coating step, the excess of immunocapture reagent was washed away by 3 PBS washing steps as illustrated for storage buffer removal. After the last washing step, magnetic beads were resuspended in 150 µL per number of samples of PBS (ex., per 10 samples: 1.5 mL of PBS) and immediately used or stored in refrigerated conditions for up to 1 week.

### 4.6. Immunocapture Protocol

Immunocapture was performed in Thermo KingFisher 96 Deepwell plates (Thermo Fisher Scientific, Waltham, MA, USA). Keeping the plate on a magnetic holder, 350 µL of PBS was dispensed in each well, followed by 150 µL of coated magnetic beads and 3 µL of sample. The immunocapture step was carried out on a Thermomixer (Eppendorf, Hamburg, Germany) for 120 min at 22 °C, 1000 rpm shaking. After the immunocapture step, non-specific IgG was removed from the magnetic beads with a double-wash step using PBS 1× (500 μL) using the ThermoFisher King Fisher automated platform. The beads were then washed with LC-MS H_2_O (500 μL) to remove excess PBS 1× salts and were eventually released in 120 μL LC-MS H_2_O for downstream applications.

### 4.7. Digestion by Rat Tritosome Protocol

Digestion protocol setup for digestion time, lysosome concentration, and buffer conditions has been based on the previous work of Colangelo et al. [19]. Digestion by Rat tritosomes was performed on the magnetic beads in the KingFisher 96 Deepwell plate. To remove the 120 µL of water used for elution, the plate was maintained on a magnetic plate holder so as not to remove the magnetic beads carrying the target ADC. After 120 µL of water removal, 50 µL of digestion mix containing Internal Standard were added in each well, excluding control blanks. A different mix containing water instead of the aqueous IS solution was applied in the control blank wells, Digestion mix composition per sample was 37.5 µL of Internal Standard solution (IS payload A 50 ng/mL, IS payload B 50 ng/mL, in water), 5 µL of DTT 20 mM, 5 µL of catabolism buffer (Sodium Acetate, pH 5.0) and 2.5 µL of Tritosome fraction mix, for a total volume of 50 µL. For control blank samples, the Internal standard solution was replaced with LC-MS water. After the digestion mix dispensation, the plate was closed with an adhesive sealer and removed from the magnetic holder, carefully resuspending the beads. Digestion was carried out in a Thermomixer with ThermoTop^®^ at 37 °C, 1000 rpm, overnight (19 h +/− 1 h).

### 4.8. Protein Precipitation and Concentration

After the overnight digestion, the KingFisher 96 Deepwell plate was moved to the magnetic holder to immobilize the magnetic beads, and 40 µL of the digestion mix was moved to a new low-binding 2 mL Deepwell 96-well plate (QuanRecovery with MaxPeak, 700 µL, p/n 41121806 by Waters, Milford, MA, USA). In the new plate, 320 µL of precipitation reagent (Acetonitrile:Methanol 95:5) was added to each well and mixed on a Thermomixer (T = 22 °C, 1000 rpm) for 5 min to precipitate protein residuals. After the mixing step, the plate was centrifuged at 3000 rpm for 10 min (T = 4 °C). After the centrifugation step, 300 µL of supernatant was moved to a new 96-well plate, and the organic solvent was evaporated using a GenVac evaporator (Genvac, SP Scientific, Warminster, PA, USA), following a 70-min total time program (program: HPLC, Time to Final Stage: 30 min, Final Stage Time: 40 min, max T limit= 70 °C). After evaporation, 150 µL of Phase A (H_2_O:ACN 95:5, 0.1% Formic Acid) was pipetted into each well for analyte resuspension and mixed on a Thermomixer for 30 min at 10 °C, 1000 rpm, before LC-MS detection.

### 4.9. LC-MS Detection

Liquid chromatography separation was performed on a SCIEX Exion LC system AD Series (SCIEX, Framingham, MA, USA), equipped with a Phenomenex Kinetex 2.6 µm PS C18 100 Å 2.1 × 100 mm column (Phenomenex, Torrance, CA, USA). Autosampler temperature was set to 10 °C. Separation was achieved using 1 min isocratic in 100% Phase A (H_2_O:ACN 95:5, 0.1% Formic Acid) followed by a 3.5 min gradient to 40% Phase B (ACN:H_2_O 95:5, 0.1% Formic Acid) with a flow of 0.6 mL/min. After separation, 100% Phase B was reached in 0.5 min to wash the column, and maintained for 1.5 min. After the washing step, a multi-step process to avoid carryover was applied, going to 0% Phase B in 0.5 min, then back to 100% Phase B in 0.5 min, and again 0% Phase B in 0.5 min. The column was then equilibrated for 2 min, for a total chromatography run time of 10 min. The column oven temperature was set to 40 °C for the entire chromatographic run duration. The needle valve was rinsed internally and externally using a sequence of strong washes (H_2_O:ACN:MeOH:2-Propanol 25:25:25:25) and Phase A during the wash phase of the gradient in every chromatography run. LC flow was directed to waste by the diverter valve from injection to min 2, then to the Mass Spectrometer from min 2 to 5, and again to waste during washing and equilibrating steps. Mass Spectrometry detection was performed using a SCIEX 6500+ Triple quadrupole system equipped with a Turbo Ion V ESI source operated in positive mode (Mass Spectrometer and Ion source purchased from SCIEX, Framingham, MA, USA). Detection was performed in MRM mode using dedicated periods for each analyte and optimized source and MS conditions for each analyte, payload A and B. For quantitation, the area ratio obtained by the area of each analyte multiple transitions trace and the related internal standard was used.

### 4.10. Method Validation

To verify method performance and reliability, a total of 5 analytical runs were performed to evaluate:

- Linearity
- Accuracy and Precision
- Sensitivity
- Selectivity
- Carryover
- Matrix Effect
- Recovery
- Effect of Dilution

Details, concentration levels, and number of replicates for each test are described in Table 4. Quantitative calculations and parameters such as method linearity, accuracy, and precision (intra and inter run), carryover, matrix effect, recovery, and sample dilution were quantified and calculated using the internal Laboratory Information Management System (Watson LIMS 7.7.1) (Thermo Scientific). Metadata (consisting of an integrated peak area for analytes and IS) were imported from SCIEX Analyst 1.7.3 software to LIMS, and curve regression and quantitation of spiked samples were performed in the LIMS environment. The acceptance criteria refer to internal procedures which reflect the guideline set forth in January 2023—International Council of Harmonization (ICH) guideline M10 on bioanalytical method validation and study sample analysis (Step5), reflected also in Food and Drug Administration (FDA) Guidance for Industry—Bioanalytical Method Validation.

### 4.11. Post Spike Solution Preparation for Recovery Evaluation Test and Matrix Effect

For recovery evaluation, the full protocol dilution factor of the method starting from 3 µL of samples was calculated, and 3 different solutions were prepared as 10× of the calculated absolute resulting concentration at the different QC levels. The global dilution factor for the overall protocol has been calculated as 75 fold. Solutions were prepared in Phase A, starting from 10 µg/mL stocks in H_2_O:ACN 50:50. Post spike solutions at 10× concentrations were prepared for each of the QC level, calculated on the final relative concentration of the payloads; 15 µL of each of the different 10× solutions were then spiked in 135 µL of resuspended extracted blank sample to be considered as absolute reference 100% for the recovery testing, and 15 µL of the same solutions was spiked in Phase A as reference 100% for matrix effect assay.

### 4.12. Total Antibody Quantitation

For total antibody quantitation, MSD GOLD 96-well Quickplex plates coated with streptavidin are blocked twice with 200 μL SuperBlock^®^ Blocking Buffer for each well for 5 min at RT. The plates are then washed three times with 200 μL of PBS 1× containing 0.05% Tween-20. MSD GOLD 96-well Quickplex plates are then saturated with 50 μL per well of biotinylated polyclonal goat anti-human IgG (Fc fragment-specific) at 450 rpm for 1 h at RT. After the coating step, the plates are washed three times with PBS 1×, 0.05% Tween-20, and then 50 μL of 1:100 diluted samples are pipetted and incubated on MSD plates at 450 rpm for 1 h at RT. After three washing steps with PBS 1× 0.05% Tween-20, 50 μL of SULFO-TAG reagent in PBS 1×, 0.05% Tween-20, 1% BSA is mixed in each well at 450 rpm for 1 h at RT. After the three washing steps with PBS 1× 0.05% Tween-20, 150 μL of MSD Read Buffer T 2× is added to each well, and the plate is finally read on a Meso Quickplex SQ120 (Meso Scale Discovery, Rockville, MD, USA) plate reader within 5 ± 1 min.

## Figures and Tables

**Table 1:** Main validation results for both conjugated payloads.

Validation Element	Assessment	Acceptance Criteria	Payload A	Payload B
Method Linearity	Calibration curve	%Bias within ±20.0% (±25.0% at the LLOQ)	Overall %Bias: >−8.7, <12.1	Overall %Bias: >−6.7, <9.1
		75% of all calibration samples must be within the accuracy range	100%	100%
Accuracy and Precision	Intra-run	Mean %Bias within ±20.0% (±25.0% at the LLOQ)	Mean %Bias: >−8.3, <11.3	Mean %Bias: >−13.1, <14.4
		%CV ≤ 20.0% (≤25.0% at the LLOQ)	%CV: >1.4, <17.9	%CV: >2.4, <15.9
	Inter-run	Mean %Bias within ± 20.0% (±25.0% at the LLOQ)	Mean %Bias: >−3.9, <−0.1	Mean %Bias: >−7.4, <1.5
		%CV ≤ 20.0% (≤25.0% at the LLOQ)	%CV: >4.9, <14.1	%CV: >6.8, <16.9
Sensitivity	Covered in intra-run accuracy and precision	SIGNAL to NOISE (average area LLOQ/average area Blank) > 5 LLOQ: 5 nM ADC	24.9	15.3
Sample Dilution	Effect of dilution	Exceeding sample: resulting ALOQ	Dilution 6: ALOQ	Dilution 6: ALOQ
		dilution within the calibration range: Mean %Bias within ±20.0%	Dil 22.5: 10.5% Dil 50: 7.3%	Dil 22.5: 12.8% Dil 50: 7.7%
		dilution within the calibration range: %CV ≤ 20.0%	Dil 22.5: 0.2% Dil 50: 1.8%	Dil 22.5: 1.6% Dil 50: 5.4%
Carryover	Carryover	Analyte response after ULOQ must be ≤25.0% of the LLOQ peak area in the 3 Accuracy and Precision runs (*n* = 3)	5.1%	9.1%
		IS response after ULOQ must be ≤5.0% of IS area in the Control Blank IS sample in the 3 Accuracy and Precision runs (*n* = 3) ULOQ: 1000 nM ADC	0.0	0.1
Selectivity	Matrix samples (matrix selectivity)	Analyte response in Blank samples (average) ≤ 20.0% analyte response in SSLLOQ (average)	4.0	6.5
		IS response in blank samples (average) ≤ 5.0% average IS peak area in calibration curve	0.0	0.0
	Matrix fortified with IS	Analyte response in control Blank IS ≤ 20.0% mean analyte area in SSLLOQ	2.7	9.3
Matrix Effect	Matrix Effect	AN and IS ME should be eliminated or minimized. %CV normalized IS ≤ 20.0% at each level.	SSL = AN ME: 1.04, IS ME: 1.04	SSL = AN ME: 0.98, IS ME: 0.97
			SSH =AN ME: 1.10, IS ME: 1.05	SSH =AN ME: 1.03, IS ME: 1.00
			SSL = AN %CV: 1.0, IS %CV: 3.8	SSL = AN %CV: 2.0, IS %CV: 3.1
			SSH = AN %CV: 10.0, IS %CV: 3.8	SSH = AN %CV: 1.0, IS %CV: 1.0
Recovery	Method Recovery	Analyte recovery should be consistent and reproducible.	L: 59.5% M: 62.5% H: 62.0%	L: 24.9% M: 28.2% H: 34.6%

**Table 2:** Recovery evaluation results for payload A and payload B, as absolute reference (100%), application of immunocapture and digestion (Full protocol), and digestion only (w/o immunocapture).

		**Payload A**				
**QC-Level**		**Response** **Absolute Reference (100%)**	**Response** **Full Protocol**	**Recovery (%)**	**Response** **w/o Immunocapture**	**Yield (%)**
L	Mean	1,832,349.83	1,089,799.60	59.5	430,322.33	23.5
	*n*	3	3		3	
	%CV	1.0	7.9		4.8	
M	Mean	39,150,336.17	24,454,063.73	62.5	9,257,682.97	23.6
	*n*	3	3		3	
	%CV	3.5	15.8		5.6	
H	Mean	102,123,819.23	63,292,591.87	62.0	23,984,189.77	23.5
	*n*	3	3		3	
	%CV	3.1	2.3		7.2	
Overall (%) Recovery	Mean			61.3		23.5
		**Payload B**				
**QC-Level**		**Response** **Absolute Reference (100%)**	**Response** **Full Protocol**	**Recovery (%)**	**Response** **w/o Immunocapture**	**Yield (%)**
L	Mean	1,922,602.1	478,558.0	24.9	438,528.9	22.8
	*n*	3	3		3	
	%CV	0.37	1.40		3.34	
M	Mean	37,055,462.9	10,466,628.1	28.2	8,774,767.4	23.7
	*n*	3	3		3	
	%CV	0.98	17.18		2.23	
H	Mean	95,102,277.8	32,943,762.8	34.6	21,957,249.7	23.1
	*n*	3	3		3	
	%CV	3.3	1.94		3.26	
Overall (%) Recovery	Mean			29.2		23.2

**Table 3:** Condensed applicability results for each of the tested ADCs as CV% and applicability on 5 concentration levels; *n* = 5 independent measurements.

**ADC ID**	**Payload A**									
	**LLOQ** **(5 nM ADC)**		**L** **(15 nM ADC)**		**M** **(300 nM ADC)**		**H** **(800 nM ADC)**		**ULOQ** **(1000 nM ADC)**	
	**CV %**	**BIAS**	**CV %**	**BIAS**	**CV %**	**BIAS**	**CV %**	**BIAS**	**CV %**	**BIAS**
ADC-B	10.8	15.3	5.0	−1.9	1.4	−0.7	2.0	−3.8	2.1	−6.5
ADC-C	0.8	4.9	7.3	−9.0	1.5	−2.9	1.8	−0.7	4.1	1.3
ADC-D	2.8	−4.8	1.2	−6.3	6.1	8.7	5.0	4.3	3.9	2.5
**ADC ID**	**Payload B**									
	**LLOQ** **(5 nM ADC)**		**L** **(15 nM ADC)**		**M** **(300 nM ADC)**		**H** **(800 nM ADC)**		**ULOQ** **(1000 nM ADC)**	
	**CV %**	**BIAS**	**CV %**	**BIAS**	**CV %**	**BIAS**	**CV %**	**BIAS**	**CV %**	**BIAS**
ADC-B	9.2	14.7	3.3	0.2	1.9	2.2	3.3	0.1	2.2	−2.2
ADC-C	4.3	−9.3	2.5	−7.5	1.4	0.1	2.0	0.4	2.7	0.7
ADC-D	4.1	−2.7	3.6	−0.4	2.3	4.5	1.5	3.0	2.3	0.7

**Table 4:** Method validation parameters.

Test	Parameter	Batches	Concentration Levels	*n*	Acceptance Criteria
Method Linearity	Calibration curve	Included in each run	10, additionally control blank and control blank IS	1	%Bias within ±20.0% (±25.0% at the LLOQ)
					75% of all calibration samples must be within the accuracy range
Accuracy and Precision	Intra-run	1	5 (SSLLOQ, SSL, SSM, SSH, SSULOQ)	5	Mean %Bias within ±20.0% (±25.0% at the LLOQ)
					%CV ≤ 20.0% (≤25.0% at the LLOQ)
	Inter-run	3 (2 + Intra-run)	5 (SSLLOQ, SSL, SSM, SSH, SSULOQ)	5	Mean %Bias within ±20.0% (±25.0% at the LLOQ)
					%CV ≤ 20.0% (≤25.0% at the LLOQ)
					3 runs passed. If one run failed, repeat it in a subsequent run
Sensitivity	Covered in intra-run accuracy and precision				SIGNAL/NOISE (average area LLOQ/average area Blank) > 5
Carryover	Carryover	Included in each run (Control Blank next to ULOQ calibration standard, evaluated in each run)			Analyte response after ULOQ must be ≤25.0% of the LLOQ peak area
					IS response after ULOQ must be ≤5.0% of the IS area in the Control Blank IS sample
Selectivity	Matrix samples (matrix selectivity)	Covered with SSLLOQ, Control Blank, and Control Blank IS in another test			Analyte response in Blank samples (average) ≤ 20.0% analyte response in SSLLOQ (average)
					IS response in blank samples (average) ≤ 5.0% average IS peak area in calibration curve
	Matrix fortified with IS	Covered with SSLLOQ, Control Blank, and Control Blank IS in another test			Analyte response in control Blank IS ≤ 20.0% mean analyte area in SSLLOQ
Matrix Effect	Matrix Effect	1	2 (SSL and SSH)	3	Analyte and IS ME should be removed or minimized
					%CV normalized IS ≤ 20.0% at each level
Recovery	Method Recovery	1	3 (SSL, SSM, and SSH)	3	Analyte and internal standard recovery should be consistent and reproducible
Sample Dilution	Effect of dilution	1	Stock 10,000 nM. Dilutions applied: 1:6, 1:22.5, 1:50	3	Exceeding sample: resulting ALOQ
					Dilution within the calibration range: Mean %Bias within ±20.0%
					Dilution within the calibration range: %CV ≤ 20.0%

## Data Availability

The data presented in this study are available in this article or on request from the corresponding authors. Access to some data may be subject to proprietary restrictions and requires approval by Merck KGaA, Darmstadt, Germany.

## Abbreviations

The following abbreviations are used in this manuscript:

ADC	Antibody-drug conjugate
ALOQ	Above limit of quantitation
AN	Analyte
CAD	Collision-activated dissociation gas
CE	Collison energy
CUR	Curtain gas
CV	Coefficient of variation %
CXP	Collision cell exit potential
DAR	Drug to Antibody Ratio
Dil	Dilution
DP	Declustering potential
DTT	DL-Dithiothreitol
EP	Entrance potential
ESI	Electrospray ionization
FDA	Food and Drug Administration
H	High
HPLC	High-pressure liquid chromatography
huFcRn	Human neonatal fragment crystallizable receptor
ICH	International Council of Harmonization
IgG	Immunoglobulin G
IS	Internal standard
K2EDTA	Dipotassium ethylenediaminetetraacetic acid
L	Low
LC-MS or LC-MS/MS	Liquid chromatography tandem Mass Spectrometry
LIMS	Laboratory information management system
LLOQ	Lower Limit of Quantitation
M	Medium
ME	Matrix effect
MRM	Multiple reaction monitoring
PBS	Phosphate-buffered saline
PK	Pharmacokinetics
PK/PD	Pharmacokinetic/Pharmacodynamic
QC	Quality control
RP	Reverse Phase
rpm	Round per minute
RT	Room temperature
SD	Sprague Dawley rat strain
SS	Spiked sample
SSH	Spiked sample high QC level
SSL	Spiked sample low QC level
SSLLOQ	Spiked sample lower Limit of Quantitation
SSM	Spiked sample medium QC level
SSULOQ	Spiked sample upper Limit of Quantitation
Tg32	Tg32 human FcRn transgenic mouse model
ULOQ	Upper Limit of Quantitation
vcMMAE	Valine-citrulline Mono-methyl Auristatin E
